# Supplementary material for: Eucalyptus-Mediated Synthesized Silver Nanoparticles-Coated Urinary Catheter Inhibits Microbial Migration and Biofilm Formation
Source: Nanomaterials (Basel). 2022 Nov 18;12(22):4059. doi: 10.3390/nano12224059 (PMC9699417; doi:10.3390/nano12224059)
Supplement: Supplementary file 1 [file nanomaterials-12-04059-s001.zip › nanomaterials-2008388-Supplementary.pdf]

Supplementary Material:

Eucalyptus-mediated synthesized silver nanoparticles-coated urinary catheter inhibits microbial migration and biofilm formation

Sakkarin Lethongkam[1,2,3], Supakit Paosen[2,3], Siwaporn Bilhman[1,2,3], Krittima Dumjun[2,3,4], Suttiwan Wunnoo[1,2,3], Suntree Choojit[1], Ratchaneewan Siri[5], Chalongrat Daengngam[5] and Supayang P. Voravuthikunchai[1,2,3], Tanan Bejrananda[6,*]

1 Natural Product Research Center of Excellence, Faculty of Science, Prince of Songkla University, Hat Yai, Songkhla 90110, Thailand
2 Center of Antimicrobial Biomaterial Innovation-Southeast Asia, Prince of Songkla University, Hat Yai, Songkhla 90110, Thailand
3 Science for Industry Program, Faculty of Science, Prince of Songkla University, Hat Yai, Songkhla 90110, Thailand
4 Division of Biological Science, Faculty of Science, Prince of Songkla University, Hat Yai, Songkhla 90110, Thailand
5 Division of Physical Science, Faculty of Science, Prince of Songkla University, Hat Yai, Songkhla 90110, Thailand
6 Department of Surgery, Division of Urology, Faculty of Medicine, Prince of Songkla University, Songkhla 90110, Thailand
* Correspondence: btanan@medicine.psu.ac.th

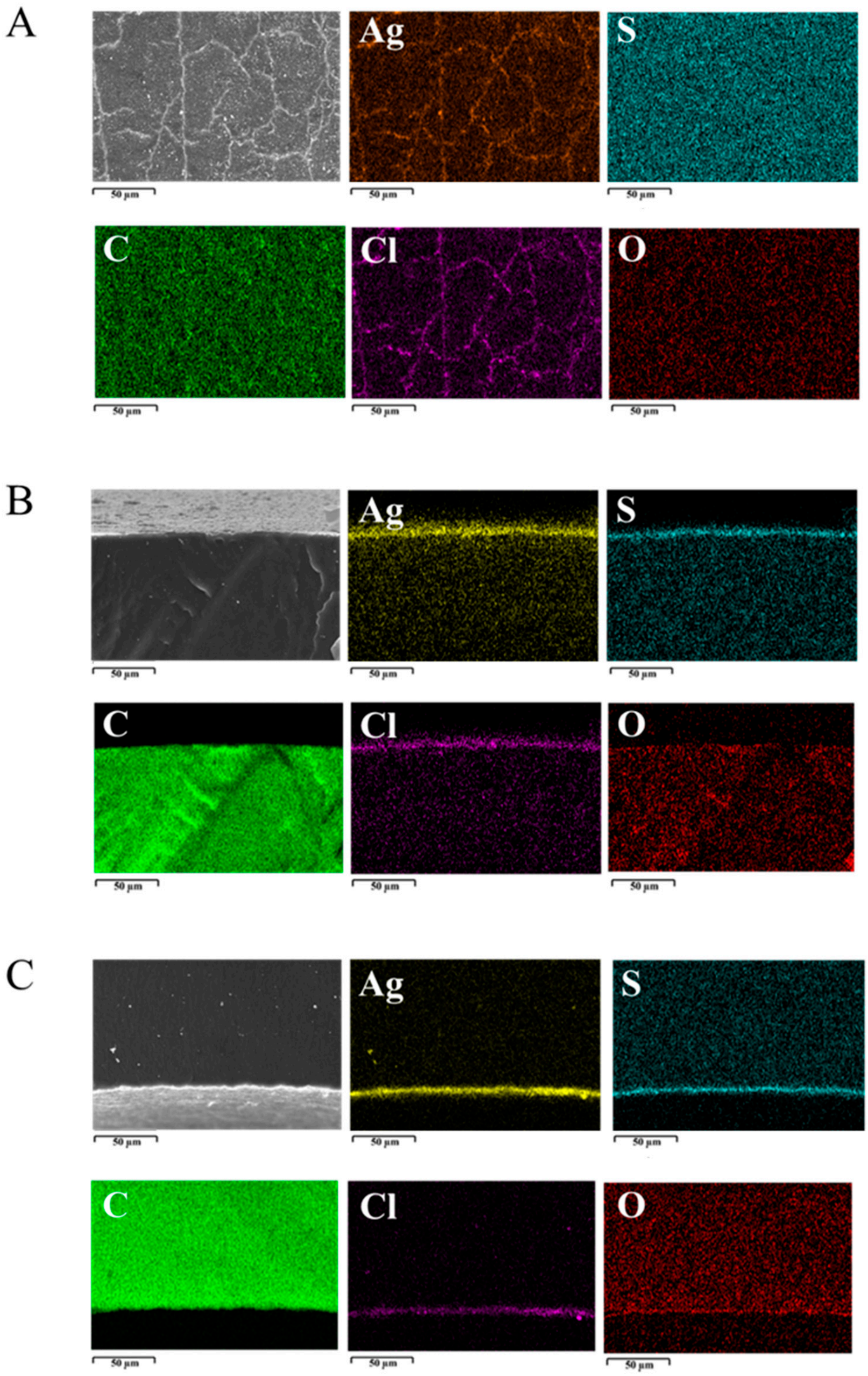

Figure S1. EDX elemental color mapping images of surface (A), cross-sectional outer (B) and inner surface (C) of AgNPs coated urinary catheters. Ag = silver, S = sulphur, C = carbon, Cl = chloride, O = oxygen
